# Safety-oriented planning of expressway truck service areas based on driver demand

**DOI:** 10.3389/fpubh.2022.976495

**Published:** 2022-08-02

**Authors:** Wenlong Ding, Yunyun Wang, Pengzi Chu, Feng Chen, Yongchao Song, Ning Zhang, Dong Lin

**Affiliations:** ^1^The Key Laboratory of Road and Traffic Engineering, Ministry of Education, Tongji University, Shanghai, China; ^2^Faculty of Civil Engineering and Geosciences, Delft University of Technology, Delft, Netherlands; ^3^School of Traffic and Transportation, Chongqing Jiaotong University, Chongqing, China; ^4^Civil and Environmental Engineering, University of California, Los Angeles, Los Angeles, CA, United States; ^5^School of Engineering, University of Aberdeen, Aberdeen, United Kingdom

**Keywords:** expressway driving safety, truck service areas, non-linear optimization model, improved genetic algorithm, service loss rate

## Abstract

The rapid development of the economy has promoted the growth of freight transportation. The truck service areas on expressways, as the main places for truck drivers to rest, play an important role in ensuring the driving safety of trucks. If these service areas are constructed densely or provide a plentiful supply of parking areas, they are costly to construct. However, if the distance between two adjacent truck service areas is very large or the number of truck parking spaces in service areas is small, the supply will fail to meet the parking needs of truck drivers. In this situation, the continuous working time of truck drivers will be longer, and this is likely to cause driver fatigue and even traffic accidents. To address these issues, this paper established a non-linear optimization model for truck service area planning of expressways to optimize truck driving safety. An improved genetic algorithm is proposed to solve the model. A case study of a 215.5-kilometers-length section of the Guang-Kun expressway in China was used to demonstrate the effectiveness of the model and algorithm. As validated by this specific case, the proposed model and solution algorithm can provide an optimal plan for the layout of truck service areas that meet the parking needs of truck drivers while minimizing the service loss rate. The research results of this paper can contribute to the construction of truck service areas and the parking management of trucks on expressways.

## Introduction

Expressways are characterized by simple road geometries where drivers usually travel for long distances at high speeds (1). With the rapid development of expressway construction, expressway freight transportation plays an important role in the whole freight transportation system. Expressway freight transportation can promote rapid development of the economy (2–4). Expressway traffic accidents are usually very serious due to the high speed of vehicles (5), and the heavy weight and large size of trucks (6–9). Therefore, greater attention should be paid to ensuring truck driving safety on expressways.

A lot of research has been done to identify the factors that contribute to truck accidents (10, 11). Among the factors involved in accidents, human factors are among the most important (12). Of the factors affecting driving performance, driver fatigue is vital, and a subject on which many studies have been conducted (13–15). Philip et al. (16) found that driver fatigue adversely and significantly impacts driver performance on driving tests. Driver fatigue is a major concern in road safety (17–19). Continuous driving without rest is a common situation for truck drivers (12), and may be aggravated by overloaded and irregular work schedules (20). Especially when driving on expressways, if the distance between service areas is too large, or there are too few truck parking spaces in the service areas, this will cause truck drivers to drive continuously for a long time. Some research has indicated that driving performance measures begin to deteriorate after 2 h of driving (21). Drivers who have driven for a long period of time without a break are more likely to experience drowsiness, which can lead to a decline in attention (22). Some research has studied the relationship between rest time and driver fatigue (14, 16, 17, 23). Accordingly, much research has been done to identify useful methods to relieve driver fatigue. Merat and Jamson (13) describe road-based measures for alleviating the symptoms of fatigue, and these include variable message signs, chevrons, and rumble strips. A rumble strip is a narrow band of built-in unevenness in the road surface that is normally placed close to the edge line or at the centerline, producing a short alerting effect if the truck diverts from the driving lane (24).

Because of the terrible potential effect of drowsy driving, many countries have put in place a lot of countermeasures, such as increasing public awareness and education, developing traffic safety policies, and using advanced vehicle technologies. In addition, installation of rest areas alongside freeways or expressways provides drivers with opportunities to eat, rest and refuel their vehicles and this enhances the comfort and convenience of drivers (especially considering the limited number of access points in an expressway system), and reduces fatigue-related crashes (1, 25, 26). The installation of proper heavy-vehicle rest area facilities is essential for safe and efficient interstate freight transport (26). In South Korea, supplemental rest areas have been installed on expressway systems to allow road users to rest; these are small-sized resting facilities located between the larger regular rest areas and have proven to be cost-effective (1). Rahman and Kang (15) found that the drowsy driving advisory, which is an engineering countermeasure designed to reduce the likelihood of drowsy-driving crashes, could significantly reduce driving crashes when cooperated with rest area facilities.

The service area is an important part of the expressway and plays an important role in traffic safety. In addition to the physiological characteristics of truck drivers, trucks traveling on expressways also have needs such as refueling and maintenance. Service areas can meet the demands of both drivers and trucks, such as having a rest, catering, refueling, and making repairs. Increasing the total rest-break duration and taking more rest breaks can reduce fatigue-related crash risk (20). Therefore, resting in the service area can help reduce driver fatigue, thus benefiting expressway safety.

Many scholars have studied the issues related to the expressway service area setting, management, etc., Jian-Wei and Yi (27) proposed a transportation potential calculation model for the service area of the expressway using transportation potential theory. Jiang and Hou (28) studied the characteristics of expressways to make the spatial planning scale of the service area reasonable and appropriate, which can not only meet the needs of customers, but also can save investment, reduce land use, and protect the environment. Koo et al. (29) developed a decision support system to determine the optimal size of the service areas of the expressways. Wu et al. (30) proposed a decision-making framework for ensuring the validity of the site selection of the expressway service area photovoltaic. By analyzing the operating data of the expressway service area, Wang et al. (31) predicted the transient population in the service area of the expressway using the Long Short-Term Memory method. Shen et al. (32) provided a theoretical basis for layout optimization and evaluation for the expressway service areas based on the economic and social adaptability. Zhao et al. (33) developed a traffic flow prediction model to improve the expressway service area management ability. Zhi et al. (34) conducted research on the expressway intelligent service area to summarize the characteristics, analyze the existing problems and elaborate the key technologies of the expressway intelligent service area.

Parking spaces are an important part of the service areas on expressways. There is a strong relationship between the number of parking spaces in the service areas and the driving safety of truck drivers. Based on the traffic volume, the distance between service areas and the parking rate, the appropriate number of parking spaces can be determined (35). The parking rate can be forecast through methods such as field surveys, BP neural networks (36), and genetic algorithms (32).

Nowadays, problems still exist in the planning of truck service areas in many countries worldwide, resulting in problems such as unbalanced service area spacing and insufficient numbers of parking spaces. At present, many countries in the world still lack a standardized method for calculating the spacing of service areas and the number of parking spaces for trucks in service areas. This study focuses on the distance between truck service areas and the number of parking spaces truck drivers need to ensure driving safety.

The remainder of this paper is organized as follows: Section Problem statement and notation presents the problem statement and variable definitions. Section Mathematical model provides the mathematical model for truck service area planning. Section Solution algorithms presents an improved genetic algorithm to solve the model. The model and algorithm are numerically validated in Section Numerical example, based on an expressway route in Yunnan province, China. Section Conclusion provides research conclusions and recommendations for further research.

## Problem statement and notation

### Problem statement

In the context of expressways with mixed passenger and freight traffic in China, Sha (37) analyzed the existing problems and their causes from the perspectives of expressway traffic safety, operation efficiency, and travel quality. In addition, Sha (37) also believes that the construction of the expressway passenger and freight systems is very necessary to improve the operational efficiency and traffic safety levels of expressways.

This study provides a mathematical method to determine the appropriate distance between service areas and the number of truck parking spaces in service areas, based on the division of construction of the expressway passenger and freight systems. The assumptions in this paper are as follows:

1. Truck drivers will choose to rest in the service area after driving continuously on the expressway for a long period of time;
2. The vehicles are traveling at the same constant speed;
3. After the trucks arrive at the expressway service area, they form a single line;
4. When trucks arrive at an expressway service area, they will leave directly if there are no available parking spaces.

### Notations

The notations used in this paper are summarized in Table 1.

**Table 1:** Notation.

**Set**	
*I*	The set of service area, and *i* ∈ *I*
**Parameters**	
*r*_*i*_	Parking rate
δ_*i*_	Value of the traffic flow on the section where the service area *i* is
$\delta_{i}^{l}$	Number of light trucks included in δ_*i*_
$\delta_{i}^{m}$	Number of medium trucks included in δ_*i*_
$\delta_{i}^{h}$	Number of heavy trucks included in δ_*i*_
$\delta_{i}^{w}$	Number of long-wheelbase trucks included in δ_*i*_
$\delta_{i}^{c}$	Number of container trucks included in δ_*i*_
λ_*i*_	Number of trucks entering the service area *i* during the peak hour
*P*_*x*_*i*__(*t*)	Service loss rate in the service area *i*
*P*_max_	Maximum value of service loss rate
*T*	Maximum safety time for continuous driving
τ	Time of rest in the period *T*
*v*	Average speed of the truck
μ	Number of trucks that can be served by one parking space per unit time
ρ_*i*_	Ratio of the arrival trucks in a service area to the number of trucks that can be served by all parking spaces per unit time
*Q*_max_	Maximum number of service areas
*Q*_min_	Minimum number of service areas
*d*_max_	Maximum distance between adjacent service areas on expressways
*d*_min_	Minimum distance between adjacent service areas on expressways
*d*	Length of expressway section
**Decision variables**	
*z*	Sum of parking spaces needed for truck drivers
*Q*	Number of service areas
ε	0,1 variables
*x*_*i*_	Total number of parking spaces for all kinds of trucks in service area *i*
$x_{i}^{l}$	Number of light truck parking spaces included in *x*_*i*_
$x_{i}^{m}$	Number of medium truck parking spaces included in *x*_*i*_
$x_{i}^{h}$	Number of heavy truck parking spaces included in *x*_*i*_
$x_{i}^{w}$	Number of long-wheelbase truck parking spaces included in *x*_*i*_
$x_{i}^{c}$	Number of container truck parking spaces included in *x*_*i*_
*d*_*i*_	Distance between the service area *i* and the service area *i* − 1 (*i* ≥ 2). When *i* = 1, *d*_1_ represents the distance from the first service area to the starting point. When the (*n*-1)-th service area is the last service area, *d*_*n*_ represents the distance from the last service area to the end point

## Mathematical model

### Model of service loss rate

**Figure 1:**
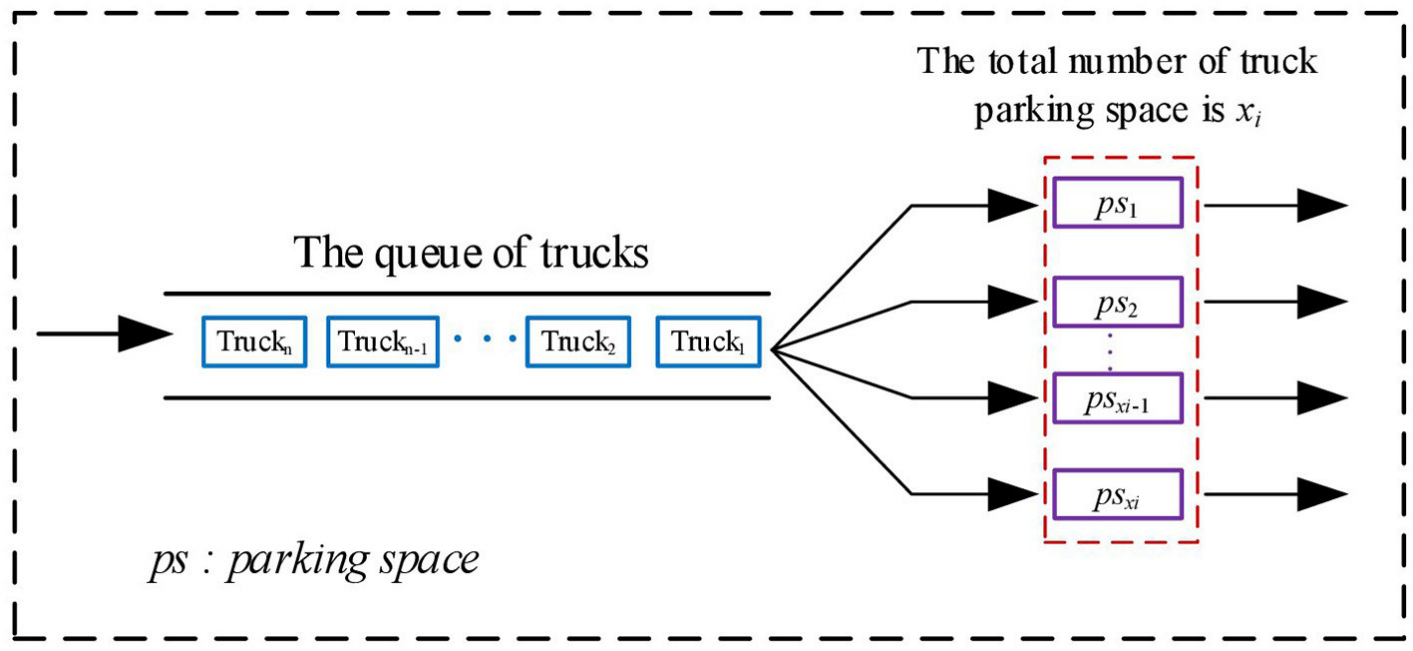
Illustration of truck parking spaces in service areas.

As shown in Figure 1, after the trucks arrive at the expressway service area, they form a single line. Therefore, it is clear that the process of parking for trucks is based on the *M*/*M*/*c*/∞/∞ queuing model.

The number of trucks entering the service area *i* can be obtained as:

(1) $$\begin{array}{r} \lambda_{i}=r_{i}\cdot \delta_{i}, \end{array}$$

where

(2) $$\begin{array}{r} \delta_{i}=\delta_{i}^{l}+\delta_{i}^{m}+\delta_{i}^{h}+\delta_{i}^{w}+\delta_{i}^{c}. \end{array}$$

Trucks parking in the expressway service areas obey the *M*/*M*/*c*/∞/∞ model.

The first *M* indicates that the trucks' arrival follows Poisson distribution. The second *M* indicates that the parking time of trucks follows exponential distribution. The symbol *c* represents the number of truck parking spaces in the service area. The first ∞ means that there is no limit to the system capacity and the second ∞ means that there is no limit to the number of trucks. Therefore, the ratio of the arriving trucks in a service area to the number of trucks that can be served by all parking spaces per unit time can be calculated as:

(3) $$\begin{array}{r} \rho_{i}=\frac{\lambda_{i}}{\mu \cdot x_{i}}. \end{array}$$

When trucks arrive at an expressway service area, drivers will leave immediately if there are no available parking spaces. This means that the parking spaces provided in the service areas cannot meet the parking needs of the truck drivers. In this case, the service loss rate can be generated. The service loss rate is an indicator used to measure the relationship between the supply of expressway service areas and the demand of truck drivers. The greater the service loss rate, the less the parking supply meets the parking demand. The smaller the value of the service loss rate, the more the truck drivers' parking needs are met. In practice, the service loss rate should be set at different values according to different situations in order to meet the parking needs of truck drivers. This will ensure driver safety and achieve the goal of saving on the construction cost of expressway service areas. The service loss rate *P*_*x*_*i*__(*t*) can be calculated as follows (38) (see Appendix A):

(4) $$\begin{array}{r} P_{x_{i}}\left(t\right)=\frac{{\left(x_{i}.\rho_{i}\right)}^{x_{i}}}{x_{i}!\sum_{m=0}^{x_{i}}\frac{{\left(x_{i}.\rho_{i}\right)}^{m}}{m!}}. \end{array}$$

In this case, we determine the maximum service loss rate is *P*_max_. Therefore, the following constraint needs to be met:

(5) $$\begin{array}{r} P_{x_{i}}\left(t\right)\leq P_{\max}. \end{array}$$

### Model of the number of expressway service areas

The number of expressway service areas should be between the maximum number and the minimum number of expressway service areas. Therefore, we can deduce:

(6) $$\begin{array}{r} Q_{\min}\leq Q\leq Q_{\max}. \end{array}$$

and

(7) $$\begin{array}{r} Q_{\min}=\left[\frac{d}{d_{\max}}\right]+\varepsilon , \end{array}$$

(8) $$\begin{array}{r} Q_{\max}=\left[\frac{d}{d_{\min}}\right]+\varepsilon , \end{array}$$

where $\left[\frac{d}{d_{\max}}\right]$ represents the largest positive integer not exceeding $\frac{d}{d_{\max}}$ and $\left[\frac{d}{d_{\min}}\right]$ represents the largest positive integer not exceeding $\frac{d}{d_{\min}}$.

The value of ε can be calculated as follows:

(9) $$\begin{array}{l} \varepsilon \text{=}\{\begin{array}{ll} 0, & \frac{d}{\hat{d}}\;is\;an\text{ int}eger\\ 1, & \frac{d}{\hat{d}}\;is\;not\;an\text{ int}eger \end{array},\hat{d}=\left\{d_{\max},d_{\min}\right\} \end{array}$$

The value of $\hat{d}$ is the set of. When $\hat{d}=d_{\max}$ and $\frac{d}{\hat{d}}$ are integers, the minimum number of truck service areas is $\frac{d}{d_{\max}}$. When $\hat{d}=d_{\max}$ and $\frac{d}{\hat{d}}$ are not integers, the minimum number of truck service areas is $\left[\frac{d}{d_{\max}}\right]+1$. By (8) and (9), we can also deduce that the maximum number of truck service areas is $\frac{d}{d_{\min}}$ when $\hat{d}=d_{\min}$ and $\frac{d}{\hat{d}}$ are integers. The maximum number of truck service areas is $\left[\frac{d}{d_{\min}}\right]+1$ under the condition where $\hat{d}=d_{\min}$ and $\frac{d}{\hat{d}}$ are not integers.

### Model of truck parking rate based on driving safety

The distance between expressway service areas should reasonably consider factors such as vehicle speed, and traffic volume. If the distance between service areas is too small, the number of service areas will increase, leading to an increase in construction costs. If the distance between service areas is too large to meet the requirements of safe driving, this can cause traffic accidents. We assume that the maximum distance allowed in the service area is *d*_max_ and the minimum distance is *d*_min_. In practice, the distance between service areas should be between the maximum and the minimum, that is

(10) $$\begin{array}{r} d_{\min}\leq d_{i}\leq d_{\max}. \end{array}$$

Driving continuously for a long time can cause driver fatigue and traffic accidents. Many countries have enacted laws limiting a driver's maximum continuous driving time. For example, China has stipulated that drivers should not drive continuously for more than 4 h. When the truck driver has been driving continuously on an expressway for a long time, he or she should drive the truck into a service area for a proper rest. In this paper, we assume that *T* is the maximum value of the driver's continuous driving time. The parking probability of trucks based on driving safety can be calculated as follows (38) (see Appendix B):

(11) $$\begin{array}{l} r_{i}=\{\begin{array}{ll} \frac{d_{i+1}}{Tv-d_{i}} & d_{i+1}<Tv-d_{i}\\ 1 & d_{i+1}\geq Tv-d_{i} \end{array} \end{array}$$

### Optimization model for truck service area planning

In this section, the optimization model for truck service area planning is established. The optimization model is as follows:

(12) $$\begin{array}{r} z=\min\sum_{i=1}^{Q}x_{i}, \end{array}$$

(13) $$\begin{array}{r} \text{subject to }\left(1\right)-\left(11\right)\text{ and} \end{array}$$

(14) $$\begin{array}{r} \sum_{i=1}^{n}d_{i}=d, \end{array}$$

(15) $$\begin{array}{r} d_{n}\leq d_{\text{m}ax}, \end{array}$$

(16) $$\begin{array}{r} x_{i}\in z^{+}. \end{array}$$

The objective function of (12) is to minimize the total number of truck parking spaces in all service areas that meet the parking needs of truck drivers. Constraint (14) indicates that the distance from the first truck service area to the starting point of the research section, together with the distances between all adjacent truck service areas, and the distance from the last truck service area to the end of the research section, equals the total length of the road section. Constraint (15) ensures that the distance from the last truck service area to the end of the road section is not greater than the maximum specified distance between adjacent truck service areas. Constraint (16) specifies the domain of decision variables.

The total number of truck parking spaces in each service area can be calculated by solving the optimization model composed of Equations (12–16). In practice, due to the different types of trucks, the types of truck parking spaces required are also different. The number of parking spaces for different trucks can be further evaluated according to the following calculation method:

(17) $$\begin{array}{r} x_{i}^{l}=\left\lceil \frac{\delta_{i}^{l}}{\delta_{i}}\cdot x_{i}\right\rceil , \end{array}$$

(18) $$\begin{array}{r} x_{i}^{m}=\left\lceil \frac{\delta_{i}^{m}}{\delta_{i}}\cdot x_{i}\right\rceil , \end{array}$$

(19) $$\begin{array}{r} x_{i}^{h}=\left\lceil \frac{\delta_{i}^{h}}{\delta_{i}}\cdot x_{i}\right\rceil , \end{array}$$

(20) $$\begin{array}{r} x_{i}^{w}=\left\lceil \frac{\delta_{i}^{w}}{\delta_{i}}\cdot x_{i}\right\rceil , \end{array}$$

(21) $$\begin{array}{r} x_{i}^{c}=\left\lceil \frac{\delta_{i}^{c}}{\delta_{i}}\cdot x_{i}\right\rceil , \end{array}$$

where Equations (17–21) represent the number of light truck parking spaces, medium truck parking spaces, heavy truck parking spaces, long-wheelbase truck parking spaces, and container carrier parking spaces, respectively, in the *i-th* truck service area.

It is worth noting that the number of truck parking spaces for different trucks calculated by Equations (17)–(21) may be a decimal. However, the actual number of truck parking spaces can only be an integer. In order to meet the parking needs of truck drivers, this paper rounds up the calculation results of different parking spaces. For example, θ represents the smallest integer greater than θ.

## Solution algorithms

**Figure 2:**
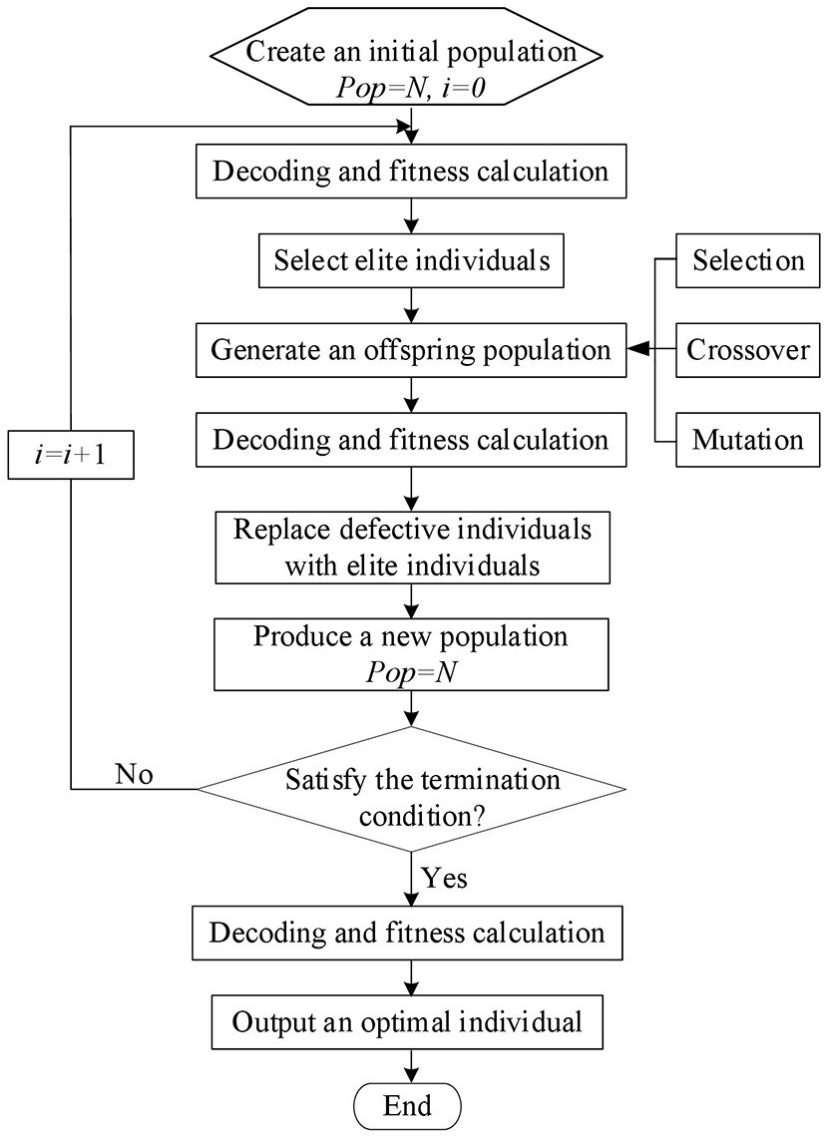
Workflow chart of genetic algorithm with the elite retention strategy.

Genetic Algorithm (GA) optimizes the feasible solution based on the idea of evolution. Traditional genetic algorithms only use three genetic operators: crossover, mutation, and selection, and this makes it difficult to converge to the global optimal solution and may even destroy the optimal individual. The optimal retention strategy is to copy one or more optimal individuals (elites) in the evolution process directly to the next generation for retention. This strategy can make the genetic algorithm converge globally. Figure 2 is a working flow chart of the genetic algorithm with an elite retention strategy. The specific steps are as follows:

Step 0: A population that fits the set coding range and population number is randomly chosen as the initial population, and the fitness of each individual in the initial population is calculated. This is the first step.
Step 1: Select elite individuals based on fitness value, and use the roulette wheel method to select people from the current population. Perform crossover operations on the selected individuals to generate new individuals. Then, on the new individuals, perform mutation operations, and finally, generate offspring populations.
Step 2: Determine the offspring population's individual fitness.
Step 3: Sort the population of offspring based on their individual fitness. Then delete bad individuals and select elite individuals. As a result, a new population is created to meet the population size.
Step 4: Determine whether the constraint condition for the algorithm's end state is satisfied. If not, go to step 2.

In the process of solving the model by using the genetic algorithm, each individual corresponds to a solution. The chromosome code in this paper includes two parts. The first part is the code for the distance between service areas, and the second part is the code for the number of parking spaces in each service area. The length of the code equals the number of decision variables.

In addition, this paper uses the roulette wheel method to select individuals, and uses a random crossover method to randomly exchange the same position codes of two individuals. The position and number of crossover and mutation operations are set to random selection. This method is better than the single-point crossover or mutation method because it helps to make new people and improve the algorithm.

## Numerical example

### Data settings

In this section, we use truck traffic flow data for a section of the Guang-Kun expressway in China to verify the effectiveness of the model and algorithm.

**Figure 3:**
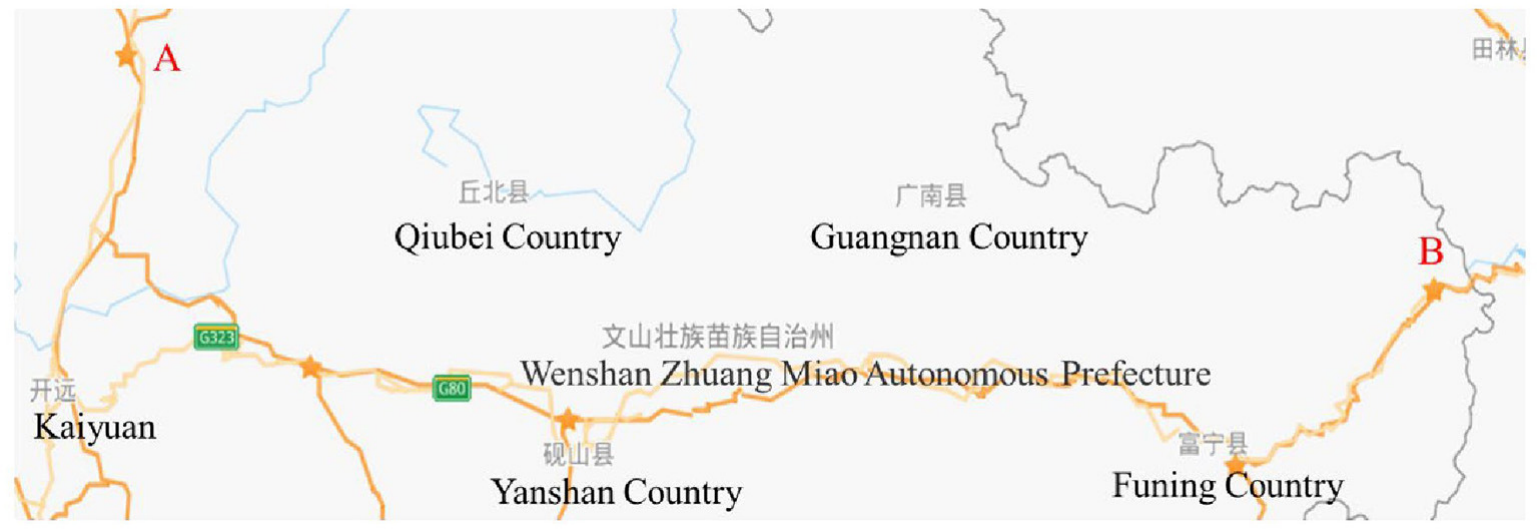
Study section of the Guang-Kun expressway in China.

As shown in Figure 3, the study section of the Guang-Kun expressway is from A to B, and the distance between A and B is about 215.5 kilometers. We collected the truck traffic flow data from April to November 2018. Analysis of the data indicates that the greatest traffic flow of trucks occurs in May. Therefore, we use the data for May to verify the effectiveness of the model and algorithm. The traffic flow data for trucks in May are shown in Table 2.

**Table 2:** Traffic flow data for trucks in May.

**Types of truck**	**Light truck**	**Medium truck**	**Heavy truck**	**Long-wheelbase truck**	**Container carrier**
Volume of traffic in May	13,400	10,338	7,947	37,765	741
Average volume of traffic in a day	432	333	256	1,218	24

The peak hour volume of traffic can be calculated by multiplying the daily average volume of traffic by the peak hour coefficient. Peak hour coefficients vary by area in China, but are generally between 0.11 and 0.15. According to certain authors, the peak hour coefficient should be 0.12 (38). Therefore, we use the peak hour coefficient of 0.12 in this paper. Calculations can be used to determine the volume of truck traffic during peak hours, as shown in Table 3.

**Table 3:** Volume of truck traffic at peak hour on an average day in May.

**Truck classification**	**Light truck**	**Medium truck**	**Heavy truck**	**Long-wheelbase truck**	**Container carrier**
Volume of traffic at peak hour	52	40	31	146	3

Article 62 of the “Regulation on the Implementation of the People's Republic of China's Road Traffic Safety Law” asserts that drivers of motor vehicles shall refrain from the following behaviors: driving a motor vehicle continuously for more than 4 h without stopping or stopping for <20 min (38).

Therefore, the value of *T* in this study is 4, and the value of μ is 3. We assume that the value of the average truck speed is *v* = 75*km*/*h*. Relevant research indicates that the distance between service areas should be between 40 and 50 kilometers (39). Therefore, the values of *d*_*min*_ and *d*_*max*_ in this paper are set to be 40 and 50, respectively. In addition, we also fix the service loss rate at 0.1.

### Calculating results for the distance between adjacent service areas problem

**Figure 4:**
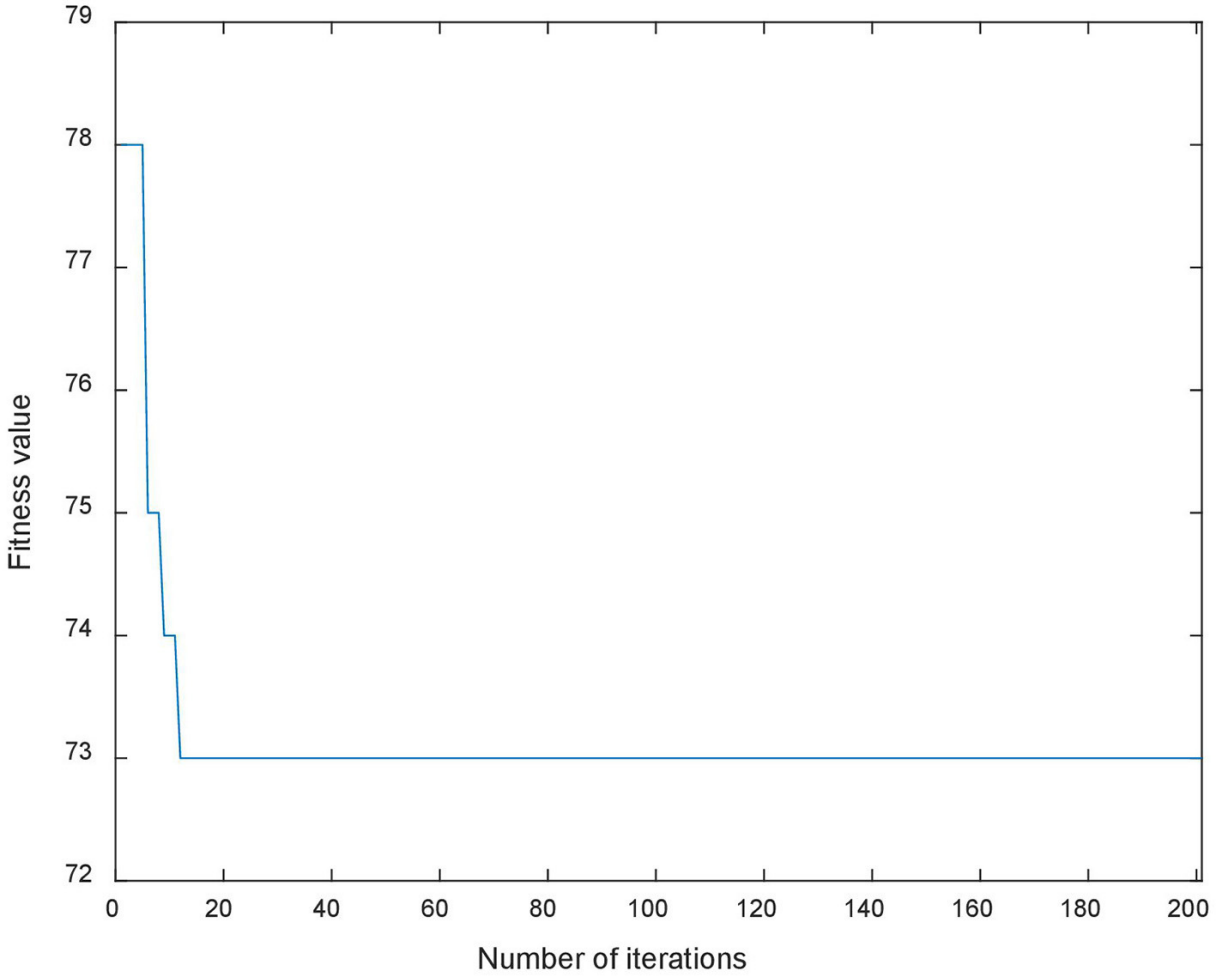
Iterative curve of fitness value.

A genetic algorithm with an elite retention strategy is adopted to solve the problem. The crossover rate and mutation rate are set at 0.8 and 0.2, respectively. The population and generation sizes of the genetic algorithm are 1,000 and 200, respectively. The algorithm is coded in MATLAB, and the change in fitness value is shown in Figure 4.

Table 4 provides the optimal results for the distance between adjacent service areas, the distance from the first service area to the start point, and the distance from the last service area to the endpoint.

**Table 4:** Calculating results of the distances.

**Distance parameters**	***d_1_***	***d_2_***	***d_3_***	***d_4_***	***d_5_***
Results of distance	41.1	49.9	42.3	41.7	40.5

From the calculation results in Table 4, we find that the distance from the starting point to the first service area is 48.7 km, the distance from the first service area to the second service area is 41.2 km, the distance from the second service area to the third service area is 45.9 km, the distance from the third service area to the fourth service area is 41.4 km, and the distance from the fourth service area to the end of the road section is 38.3 km.

### Calculating results for the number of parking spaces in service areas

Table 5 shows the calculation results for the number of parking spaces in each service area.

**Table 5:** Numbers of parking spaces in different service areas.

**Service area**	***x*_1_**	***x*_2_**	***x*_3_**	***x*_4_**
Calculation results of total parking spaces	20	18	18	17

The calculation results in Table 5 show that the total numbers of optimal truck parking spaces in the first, second, third, and fourth service areas are 18, 19, 18, and 16, respectively.

From the calculation result shown in Table 5, according to Equations (17)–(21), the numbers of parking spaces for light trucks, medium trucks, heavy trucks, long-wheelbase trucks, and container carriers in each service area can be calculated. The calculation results are shown in Table 6.

**Table 6:** Numbers of different parking spaces in the different service areas.

**Variables**	**Total number of parking spaces**	**Variables of different types of parking spaces**	**Coefficients**	**Decimal solutions**	**Integer solutions**
*x*_1_	20	$x_{1}^{l}$	52/272	3.82	4
		$x_{1}^{m}$	40/272	2.94	3
		$x_{1}^{h}$	31/272	2.78	3
		$x_{1}^{w}$	146/272	10.7	11
		$x_{1}^{c}$	3/272	0.22	1
*x*_2_	18	$x_{2}^{l}$	52/272	3.44	4
		$x_{2}^{m}$	40/272	2.65	3
		$x_{2}^{h}$	31/272	2.05	3
		$x_{2}^{w}$	146/272	9.66	10
		$x_{2}^{c}$	3/272	0.2	1
*x*_3_	18	$x_{3}^{l}$	52/272	3.44	4
		$x_{3}^{m}$	40/272	2.65	3
		$x_{3}^{h}$	31/272	2.05	3
		$x_{3}^{w}$	146/272	9.66	10
		$x_{3}^{c}$	3/272	0.2	1
*x*_4_	17	$x_{4}^{l}$	52/272	3.25	4
		$x_{4}^{m}$	40/272	2.5	3
		$x_{4}^{h}$	31/272	1.94	2
		$x_{4}^{w}$	146/272	9.13	10
		$x_{4}^{c}$	3/272	0.19	1

We take the first service area as an example to discuss the results in Table 6. In Table 6 the total number of all parking spaces in the first service area is 20. In addition, the total number of all trucks is 272, including 52 light trucks, 40 medium trucks, 31 heavy trucks, 146 long-wheelbase trucks, and 3 container carriers. Therefore, the coefficient of $x_{1}^{l}$ can be obtained as $\frac{52}{272}$. Similarly, the values of the coefficients of $x_{1}^{m}$, $x_{1}^{h}$, $x_{1}^{w}$, and $x_{1}^{c}$ can be obtained as $\frac{40}{272}$, $\frac{31}{272}$, $\frac{146}{272}$, and $\frac{3}{272}$, respectively. The number of parking spaces for light trucks is $20\times \frac{52}{272}=3.82$. According to Equation (17), the integer solution of the number of parking spaces for light trucks in the first service area is ⌈3.82⌉ = 4. Similarly, the numbers of parking spaces for medium trucks, heavy trucks, long-wheelbase trucks, and container carriers are 3, 3, 11, and 1, respectively.

## Conclusion

This study established a non-linear optimization model for expressway truck service area planning to meet driver demand for the purpose of increasing driving safety. A genetic algorithm with an elite retention strategy was proposed to solve the model. The major contributions of this study are as follows:

1. The provided model and the algorithm can provide approximate optimal strategy planning for expressway truck service areas required for driving safety. The model can estimate not only the optimal values of the distances between service areas, but also the optimal numbers of different truck parking spaces in the service areas.
2. The modeling method proposed in this study is not limited to the studied case. It can be used for planning many expressway service areas by adjusting model parameters.
3. The study results can assist in the planning of expressway truck service areas, to ensure the driving safety of truck drivers and save on the construction cost of service areas.

Based on the study results of this paper, future research is recommended as follows:

1. This paper does not consider mixed-traffic flow conditions for expressway service area planning to increase driving safety. Note that the speed differences for different vehicles should be considered when studying expressway service area planning under mixed-traffic flow conditions.
2. The setting of the number of truck parking spaces in the service area of the expressway is also affected by the truck volume. Further revision of the models proposed in this paper according to the truck volume changes is one of the key contents of future research.
3. This paper studied the planning of service areas with particular regard to driving safety. However, the factors that affect the planning of expressway service areas are complex, and these include factors such as driving behaviors, and the services provided in expressway service area. Future models involving more factors that affect the planning of expressway service areas can be developed based on the generalized model proposed in this study.

## Data availability statement

The original contributions presented in the study are included in the article/Supplementary material, further inquiries can be directed to the corresponding author/s.

## Author contributions

WD: conceptualization, methodology, and writing—original draft. YW: writing—original draft. PC: software. FC: conceptualization, formal analysis, funding acquisition, and review. YS: data curation. NZ: writing—review and editing. DL: conceptualization and writing—review and editing. All authors contributed to the article and approved the submitted version.

## Funding

This study was supported by the National Natural Science Foundation of China (51978522).

## Conflict of interest

The authors declare that the research was conducted in the absence of any commercial or financial relationships that could be construed as a potential conflict of interest.

## Publisher's note

All claims expressed in this article are solely those of the authors and do not necessarily represent those of their affiliated organizations, or those of the publisher, the editors and the reviewers. Any product that may be evaluated in this article, or claim that may be made by its manufacturer, is not guaranteed or endorsed by the publisher.
