# Supplementary material for: Safety-oriented planning of expressway truck service areas based on driver demand
Source: Front Public Health. 2022 Aug 2;10:976495. doi: 10.3389/fpubh.2022.976495 (PMC9379340; doi:10.3389/fpubh.2022.976495)
Supplement: Supplementary file 1 [file Presentation_1.pdf]

**Appendix A. Proof of Eq. (4)**

For the i-th service area, the arrival of trucks obeys the Poisson distribution of parameter $\lambda_i$, and the residence time obeys the negative exponential distribution of parameter $\mu$. The $M/M/c/\infty/\infty$ queuing model is shown in Fig.1.

As there is no difference in parking spaces, so $\mu_1 = \mu_2 = \mu_3 = ... = \mu_{x_i} = \mu$.

There are $n$ trucks in the service area at time $t + \Delta t$, which can be divided into four cases as shown in Table 7.

"Insert Table 7 here"

The probabilities are:

$$p_n(t)(1-\lambda_i\Delta t)(1-n\mu\cdot\Delta t) \tag{A.1}$$

$$p_n(t)(1-\lambda_i\Delta t)(1-n\mu\cdot\Delta t) \tag{A.2}$$

$$p_{n-1}(t)\lambda_i\Delta t\cdot[(1-(n-1)\mu\cdot\Delta t)] \tag{A.3}$$

$$p_n(t)\lambda_i\Delta t\cdot(n\mu\cdot\Delta t) \tag{A.4}$$

When $n$=0. By Eqs. (A.1), (A.2) and (A.4), we have

$$P_0(t+\Delta t) = P_0(t)[1-\lambda_i\cdot\Delta t] + P_1(t)(1-\lambda_i\Delta t)\cdot(\mu\cdot\Delta t) + o(\Delta t) \tag{A.5}$$

Because of $(\Delta t)^2 = 0$, and:

$$\frac{dP_0(t)}{dt} = \frac{P_0(t+\Delta t) - P_0(t)}{\Delta t} = P_1(t)\cdot\mu - P_0(t)\lambda_i + \frac{o(\Delta t)}{\Delta t} \tag{A.6}$$

Because of

$$\lim_{\Delta t\to 0}\left(\frac{o(\Delta t)}{\Delta t}\right) = 0 \tag{A.7}$$

We denote

$$\frac{dP_1(t)}{dt} = 0 \tag{A.8}$$

By Eqs. (A.6), (A.7) and (A.8), we have

$$P_1(t)\cdot\mu - P_0(t)\lambda_i = 0 \quad (n=0) \tag{A.9}$$

When $1 \le n \le x_i - 1$

$$\begin{aligned} P_n(t+\Delta t) = {} & p_n(t)(1-\lambda_i\Delta t)(1-n\mu\cdot\Delta t) + p_{n+1}(t)(1-\lambda_i\Delta t)(n+1)\mu\cdot\Delta t \\ & + p_{n-1}(t)\lambda_i\Delta t\cdot[(1-(n-1)\mu\cdot\Delta t)] + p_n(t)\lambda_i\Delta t\cdot(n\mu\cdot\Delta t) \end{aligned} \tag{A.10}$$

$$\begin{aligned} P_n(t+\Delta t) &= P_n(t)[1-n\mu\cdot\Delta t - \lambda_i\cdot\Delta t] + P_{n-1}(t)\cdot\lambda_i\Delta t + P_{n+1}(t)(1+n)\mu\cdot\Delta t + o(\Delta t) \\ &= P_n(t)[1-(n\mu+\lambda_i)\Delta t] + P_{n-1}(t)\cdot\lambda_i\Delta t + P_{n+1}(t)(1+n)\mu\cdot\Delta t + o(\Delta t) \end{aligned} \tag{A.11}$$

Thus

$$\frac{P_n(t+\Delta t)-P_n(t)}{\Delta t}=\lambda_i P_{n-1}(t)+(1+n)\mu P_{n+1}(t)-(n\mu+\lambda_i)P_n(t)+\frac{o(\Delta t)}{\Delta t} \tag{A.12}$$

$$\frac{dP_n(t)}{dt}=\frac{P_n(t+\Delta t)-P_n(t)}{\Delta t}=\lambda_i P_{n-1}(t)+(1+n)\mu P_{n+1}(t)-(n\mu+\lambda_i)P_n(t)+\frac{o(\Delta t)}{\Delta t} \tag{A.13}$$

By Eqs. (A.7), (A.8) and (A.13), we have

$$\lambda_i P_{n-1}(t)+(1+n)\mu P_{n+1}(t)-(n\mu+\lambda_i)P_n(t)=0 \quad (1\le n\le x_i-1) \tag{A.14}$$

In view of (A.9), when $n=0$

$$P_1(t)=(\frac{\lambda_i}{\mu})P_0(t) \tag{A.15}$$

In view of (A.14), when $n=1$

$$\lambda_i P_0(t)+2\mu P_2(t)-(\mu+\lambda_i)P_1(t)=0 \tag{A.16}$$

By Eqs. (A.15) and (A.16), it can be obtained:

$$P_2(t)=\frac{1}{2!}(\frac{\lambda_i}{\mu})^2 P_0(t) \tag{A.17}$$

When $n=2$, by Eq.(A.14), we can get

$$\lambda_i P_1(t)+3\mu P_3(t)-(2\mu+\lambda_i)P_2(t)=0 \tag{A.18}$$

In view of Eqs. (A.15), (A.17) and (A.18):

$$P_3(t)=\frac{1}{3!}(\frac{\lambda_i}{\mu})^3 P_0(t) \tag{A.19}$$

According to mathematical induction, we guess when $n=x_i-1$

$$P_{x_i}(t)=\frac{1}{x_i!}(\frac{\lambda_i}{\mu})^{x_i} P_0(t) \tag{A.20}$$

The proof process is as follows

When $n=x_i-3$

$$P_{x_i-2}(t)=\frac{1}{(x_i-2)!}(\frac{\lambda_i}{\mu})^{x_i-2} P_0(t) \tag{A.21}$$

When $n=x_i-2$

$$P_{x_i-1}(t)=\frac{1}{(x_i-1)!}(\frac{\lambda_i}{\mu})^{x_i-1} P_0(t) \tag{A.22}$$

When $n=x_i-1$, by Eq. (A.14), we have

$$\lambda_i\cdot P_{x_i-2}(t)+x_i\cdot\mu P_{x_i}(t)-[(x_i-1)\mu+\lambda_i]P_{x_i-1}(t)=0 \tag{A.23}$$

By Eqs. (A.21), (A.22) and (A.23), we can obtain

$$
\begin{aligned}
x_i \cdot \mu P_c(t) &= [(x_i-1)\mu+\lambda_i]P_{x_i-1}-\lambda_i P_{x_i-2} \\
&= [(x_i-1)\mu+\lambda_i]\frac{1}{(x_i-1)!}(\frac{\lambda_i}{\mu})^{x_i-1}P_0(t)-\lambda_i\frac{1}{(x_i-2)!}(\frac{\lambda_i}{\mu})^{x_i-2}P_0(t) \\
&= \frac{\mu}{(x_i-2)!}(\frac{\lambda_i}{\mu})^{x_i-1}P_0(t)+\frac{\lambda_i}{(x_i-1)!}(\frac{\lambda_i}{\mu})^{x_i-1}P_0(t)-\frac{\lambda_i}{(x_i-2)!}(\frac{\lambda_i}{\mu})^{x_i-2}P_0(t) \\
&= \frac{\lambda_i}{(x_i-2)!}(\frac{\lambda_i}{\mu})^{x_i-2}P_0(t)+\frac{\lambda_i}{(x_i-1)!}(\frac{\lambda_i}{\mu})^{x_i-1}P_0(t)-\frac{\lambda_i}{(x_i-2)!}(\frac{\lambda_i}{\mu})^{x_i-2}P_0(t) \\
&= \frac{\lambda_i}{(x_i-1)!}(\frac{\lambda_i}{\mu})^{x_i-1}P_0(t)
\end{aligned}
\tag{A.24}
$$

Using Eq. (A.24), we have

$$
\begin{aligned}
P_{x_i}(t) &= \frac{1}{x_i \cdot \mu}\frac{\lambda_i}{(x_i-1)!}(\frac{\lambda_i}{\mu})^{x_i-1}P_0(t) \\
&= \frac{1}{x_i!}(\frac{\lambda_i}{\mu})^{x_i}P_0(t)
\end{aligned}
$$

(A.20) is proved.
Therefore we can derive

$$
P_n(t) = \frac{1}{n!}(\frac{\lambda_i}{\mu})^n P_0(t) \quad (1 \le n \le x_i) \tag{A.25}
$$

According to (A.25), it can be derived

$$
\begin{aligned}
& P_0(t)+\sum_{n=1}^{x_i}P_{x_i}(t) \\
&= P_0(t)+\sum_{n=1}^{x_i}\frac{1}{n!}(\frac{\lambda_i}{\mu})^n P_0(t) \\
&= \frac{1}{0!}(\frac{\lambda_i}{\mu})^0 P_0(t)+\sum_{n=1}^{x_i}\frac{1}{n!}(\frac{\lambda_i}{\mu})^n P_0(t) \\
&= \sum_{n=0}^{x_i}\frac{1}{n!}(\frac{\lambda_i}{\mu})^n P_0(t) \\
&= P_0(t)\sum_{n=0}^{x_i}\frac{1}{n!}(\frac{\lambda_i}{\mu})^n
\end{aligned}
\tag{A.26}
$$

According to $\sum_{n=0}^{x_i}P_n(t)=1$, we have

$$
P_0(t) = \frac{1}{\sum_{n=0}^{x_i}\frac{1}{n!}(\frac{\lambda_i}{\mu})^n} \tag{A.27}
$$

By (A.25) and (A.27), we can obtain

$$
\begin{aligned}
P_{x_i}(t) &= \frac{1}{x_i!}(\frac{\lambda_i}{\mu})^{x_i}P_0(t) \\
&= \frac{1}{x_i!}(\frac{\lambda_i}{\mu})^{x_i}\frac{1}{\sum_{n=0}^{x_i}\frac{1}{n!}(\frac{\lambda_i}{\mu})^n}
\end{aligned}
\tag{A.28}
$$

Because of

$$\rho_i = \frac{\lambda_i}{x_i \cdot \mu} \tag{A.29}$$

Using Eqs. (A.28) and (A.29), we have

$$P_{x_i}(t) = \frac{(x_i \cdot \rho_i)^{x_i}}{x_i! \sum_{n=0}^{x_i} \frac{(x_i \cdot \rho_i)^n}{n!}}$$

Eq. (4) has been proved.

**Appendix B. Proof of Eq. (11)**

"Insert Figure 5 here"

As shown in Fig.5, there are 3 service areas, $S_{i-1}$, $S_i$ and $S_{i+1}$. The distance between $S_{i-1}$ and $S_i$ is $d_i$, and the distance between $S_i$ and $S_{i+1}$ is $d_{i+1}$. Assuming the time when the truck arrives at the service area $S_i$ is $t_i$, therefore, the time $t_{i-1}$ when the truck arrives at the service area $S_{i-1}$ can be calculated as

$$t_{i-1} = t_i - \frac{d_i}{v} \tag{B.1}$$

It can be obtained that the time for the truck driver to rest before arriving at $S_i$ may be at $t_{i-1}$ or before $t_{i-1}$. Because the continuous driving time of the truck driver cannot be greater than $T$, the next rest time of the truck driver cannot be greater than $t_i - \frac{d_i}{v} + T$ (It is worth noting that $\frac{d_i}{v} < T$). Therefore, the truck driver will have a rest during the period from $t_i$ to $t_i - \frac{d_i}{v} + T$. Suppose $\tau$ is any time between $t_i$ and $t_i - \frac{d_i}{v} + T$, which is denoted as $\tau \in [t_i, t_i - \frac{d_i}{v} + T]$ .It is defined that $f(\tau)$ is the probability density function of $\tau$ and obeys uniform distribution. We can obtain

$$f(\tau) = \frac{1}{t_i - \frac{d_i}{v} + T - t_i} = \frac{1}{T - \frac{d_i}{v}} \tag{B.2}$$

When $d_i + d_{i+1} < Tv$, the maximum value of probability of truck drivers resting in service area $S_i$ can be calculated as

$$r_i = \int_{t_i}^{t_i + \frac{d_{i+1}}{v}} f(\tau) d\tau = \frac{1}{T - \frac{d_i}{v}} \cdot (t_i + \frac{d_{i+1}}{v} - t_i) = \frac{d_{i+1}}{Tv - d_i}, \quad d_i < Tv - d_{i+1} \tag{B.3}$$

When $d_i + d_{i+1} \geq Tv$, truck drivers must have a rest in service area $S_i$. In this situation the probability of truck drivers resting in service area $S_i$ equals to 1. Therefore we can obtain

$$r_i = \begin{cases} \dfrac{d_{i+1}}{Tv - d_i} & d_{i+1} < Tv - d_i \\ 1 & d_{i+1} \geq Tv - d_i \end{cases}$$

Eq. (11) has been proved.
