# Supplementary material for: Safety-oriented planning of expressway truck service areas based on driver demand
Source: Front Public Health. 2022 Aug 2;10:976495. doi: 10.3389/fpubh.2022.976495 (PMC9379340; doi:10.3389/fpubh.2022.976495)
Supplement: Supplementary file 2 [file Presentation_2.pdf]

Appendix B. Proof of Eq. (11)

“Insert Figure 5 here”

As shown in Fig.5, there are 3 service areas, S_{i-1}, S_i and S_{i+1}. The distance between S_{i-1} and S_i is d_i, and the distance between S_i and S_{i+1} is d_{i+1}. Assuming the time when the truck arrives at the service area S_i is t_i, therefore, the time t_{i-1} when the truck arrives at the service area S_{i-1} can be calculated as

$$t_{i-1} = t_i - \frac{d_i}{v} \tag{B.1}$$

It can be obtained that the time for the truck driver to rest before arriving at S_i may be at t_{i-1} or before t_{i-1}. Because the continuous driving time of the truck driver cannot be greater than T, the next rest time of the truck driver cannot be greater than $t_i - \frac{d_i}{v} + T$ (It is worth noting that $\frac{d_i}{v} < T$). Therefore, the truck driver will have a rest during the period from t_i to $t_i - \frac{d_i}{v} + T$. Suppose τ is any time between t_i and $t_i - \frac{d_i}{v} + T$, which is denoted as $\tau \in [t_i, t_i - \frac{d_i}{v} + T]$ .It is defined that $f(\tau)$ is the probability density function of τ and obeys uniform distribution. We can obtain

$$f(\tau) = \frac{1}{t_i - \frac{d_i}{v} + T - t_i} = \frac{1}{T - \frac{d_i}{v}} \tag{B.2}$$

When $d_i + d_{i+1} < Tv$, the maximum value of probability of truck drivers resting in service area S_i can be calculated as

$$r_i = \int_{t_i}^{t_i + \frac{d_{i+1}}{v}} f(\tau) d\tau = \frac{1}{T - \frac{d_i}{v}} \cdot (t_i + \frac{d_{i+1}}{v} - t_i) = \frac{d_{i+1}}{Tv - d_i}, \quad d_i < Tv - d_{i+1} \tag{B.3}$$

When $d_i + d_{i+1} \geq Tv$, truck drivers must have a rest in service area S_i. In this situation the probability of truck drivers resting in service area S_i equals to 1. Therefore we can obtain

$$r_i = \begin{cases} \frac{d_{i+1}}{Tv - d_i} & d_{i+1} < Tv - d_i \\ 1 & d_{i+1} \geq Tv - d_i \end{cases}$$

Eq. (11) has been proved.
